# Supplementary material for: Effect of a Herbal Therapy on Clinical Symptoms of Acute Lower Uncomplicated Urinary Tract Infections in Women: Secondary Analysis from a Randomized Controlled Trial
Source: Antibiotics (Basel). 2019 Dec 7;8(4):256. doi: 10.3390/antibiotics8040256 (PMC6963539; doi:10.3390/antibiotics8040256)
Supplement: Supplementary file 1 [file antibiotics-08-00256-s001.pdf]

Supplementary Table 1. Inclusion/exclusion criteria

Inclusion criteria:

- Female outpatients aged 18–75 years
- Diagnosis of acute lower uUTI occurring for the first time or acute relapse of chronic recurrent uUTI
- Typical symptoms of cystitis (pollakisuria, dysuria and urgency)
- Bacterial count of 10^4 – 10^6 colony forming units (CFU)/mL in midstream urine
- Presence of >20 leukocytes/μL of urine measured by dipstick test
- No antibiotic treatment required according to the investigator
- Women of childbearing potential were allowed to participate only if they used a highly effective method of contraception
- Written informed consent

Exclusion criteria:

- Known hypersensitivity to any of the active substances or excipients of the study medication
- Antibiotic treatment during the past 8 days or indication for antibiotic treatment for the current infection
- Patients with trichomoniasis, chlamydiosis or gonorrhea
- Use of concomitant medication that may have an effect on the UTI, including other phytotherapeutics with similar effects, saluretics (including those in antihypertensives) or other drugs with a similar mode of action, urinary acidifying agents (e.g., Acimethin®), antibiotics, or phytotherapeutics with possible antibiotic effects
- Patients with suspected ovarian inflammation (e.g., adnexitis)
- Patients with suspected renal inflammation (e.g., pyelonephritis)
- Patients with complicated UTI (e.g., obstruction, stones, reflux)
- Patients with overactive bladder
- Patients with vegetative urogenital syndrome
- Patients who were currently participating or had participated in another clinical trial within 30 days before enrollment
- Patients in poor general condition
- Alcohol- or drug-addicted patients
- Pregnant or nursing women or women not using highly effective methods of contraception
- Patients with mental illness or no/limited legal capacity
- Patients held in an institution by legal or official order
- Patients who were not proficient in spoken or written German
- Patients with a urine bacterial count >10^6 CFU/mL were to be excluded from further participation in the study unless they specifically wished to continue treatment with the study medication.
- No contraindications against the study medication were known at the time of study protocol preparation.

Supplementary Table 2. Schedule of study assessments

	Baseline (Day 0)	After 1 day (Day 1)	After 6±1 days (Day 7)
Diagnosis, medical history, physical examination	x		
Inclusion/exclusion criteria	x		
Safety laboratory	x		x
Urine dipstick test for leukocytes and nitrite	x	x	x
Midstream urine culture (bacterial counts)	x		x
Adverse events		x	x
Documentation of symptoms (investigator / patient diary)	x	x	x
Need for antibiotic treatment		x	x
Final evaluation (investigator and patient)			x
Treatment	Continuously from Day 0 to Day 7		

ACSS Questionnaire

First visit (diagnostic form) - Part A

Time: _____:_____ Date of evaluation: / / (dd/mm/yyyy)

Please indicate whether you have had the following symptoms during the past 24 hours, and how severe they were:
(Please mark ✓ only one answer for each symptom)

			0	1	2	3
Typical	1	Frequent urination of small volumes of urine *(going to the toilet very often)*	☐ No<br>*4 or less times per day*	☐ Yes, mild<br>*5-6 times/ day*	☐ Yes, moderate<br>*7-8 times/day*	☐ Yes, severe<br>*9-10 or more times/day*
	2	Urgent urination *(a strong and uncontrollable urge to pass urine)*	☐ No	☐ Yes, mild	☐ Yes, moderate	☐ Yes, severe
	3	Feeling pain or burning when passing urine	☐ No	☐ Yes, mild	☐ Yes, moderate	☐ Yes, severe
	4	Incomplete bladder emptying after urination	☐ No	☐ Yes, mild	☐ Yes, moderate	☐ Yes, severe
	5	Pain or uncomfortable pressure in the lower abdomen *(suprapubic area)*	☐ No	☐ Yes, mild	☐ Yes, moderate	☐ Yes, severe
	6	Visible blood in your urine	☐ No	☐ Yes, mild	☐ Yes, moderate	☐ Yes, severe
					**Sum of "Typical" scores=** ☐	**points**
Differential	7	Loin *(low back)* pain*	☐ No	☐ Yes, mild	☐ Yes, moderate	☐ Yes, severe
	8	Vaginal discharge *(especially in the mornings)*	☐ No	☐ Yes, mild	☐ Yes, moderate	☐ Yes, severe
	9	Urethral discharge *(without urination)*	☐ No	☐ Yes, mild	☐ Yes, moderate	☐ Yes, severe
	10	High body temperature *(chills/fever)*	☐ No	☐ Yes, mild	☐ Yes, moderate	☐ Yes, severe
		(Please indicate ✓if measured)	≤37.5 °C	37.6-37.9 °C	38.0-38.9 °C	≥39.0 °C
		* often unilateral (on one side)			**Sum of "Differential" scores=** ☐	**points**

Quality of life

11 **Please give an overall rating of how much these symptoms, mentioned above, bothered you in the past 24 hours *(Please mark ✓ only one answer)***

- ☐ 0 Do not feel any discomfort *(No symptoms at all. Felt as good as usual)*
- ☐ 1 Feeling little discomfort *(Feeling somewhat worse than usual)*
- ☐ 2 Feeling moderate discomfort *(Feeling quite bad)*
- ☐ 3 Feeling extreme discomfort *(Feeling terrible)*

12 **Please choose the number, which most closely describes your normal work/everyday activities were affected by your symptoms, mentioned above, in the past 24 hours *(Please mark ✓ only one answer)***

- ☐ 0 Not affected at all *(Carrying out usual daily activities)*
- ☐ 1 Mildly affected *(Able to carry out daily activities with some discomfort)*
- ☐ 2 Moderately affected *(Only able to carry out daily activities with significant effort)*
- ☐ 3 Extremely affected *(Almost impossible to carry out daily activities)*

13 **Please indicate, how much your social activities were affected by your symptoms, mentioned above, in the past 24 hours *(Please mark ✓ only one answer)***

- ☐ 0 Not affected at all *(Able to enjoy normal social activities)*
- ☐ 1 Mildly affected *(Not able to do some social activities)*
- ☐ 2 Moderately affected *(Only able to do a few social activities)*
- ☐ 3 Extremely affected *(Not able to do any social activity - symptoms keep me a 'prisoner' in my home)*

Sum of "QoL" scores= ☐ **points**

Additional

14	**Please indicate whether you have the followings today:**		
	Menstruation *(women's monthly period)*?	☐ No	☐ Yes
	Premenstrual symptoms?	☐ No	☐ Yes
	Symptoms of the menopause ?	☐ No	☐ Yes
	Are you pregnant?	☐ No	☐ Yes
	Do you have diabetes mellitus *(sugar diabetes)*?	☐ No	☐ Yes

STOP **Please do not forget to return completed questionnaire back to your physician**

Thank you for cooperation

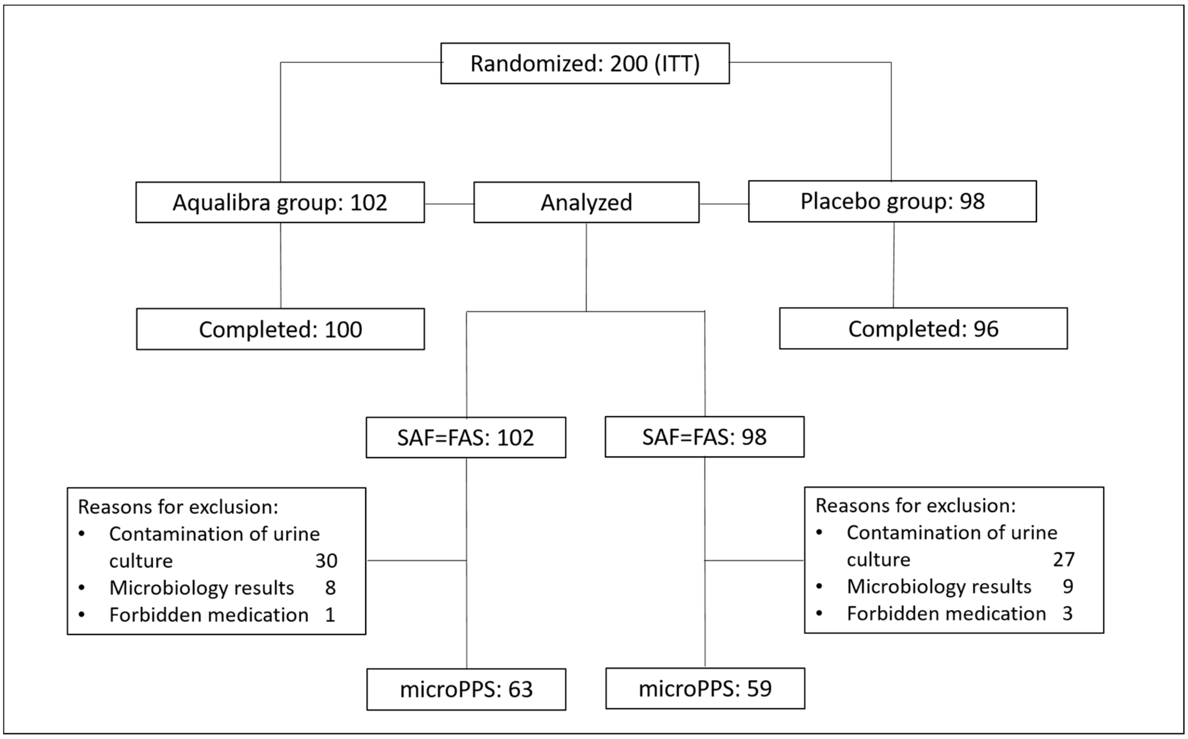

Supplementary Figure 1. Disposition of patients. FAS: full analysis set; ITT: intention-to-treat; microPPS: per-protocol set (patients with evaluable microbiologic data: patients with a bacterial count ≥ 10^4 CFU/mL at the time of inclusion, whose urine cultures were not contaminated and who did not take any concomitant medication that could interfere with urine culture); SAF: safety analysis set.

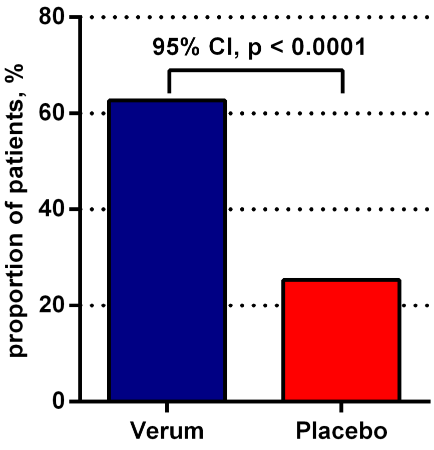

Supplementary Figure 2. Secondary analysis of the primary endpoint using microbiologic response criteria as defined by current draft guidelines (i.e., a bacterial count reduction from $\geq 10^5$ to $<10^3$ CFU/mL).

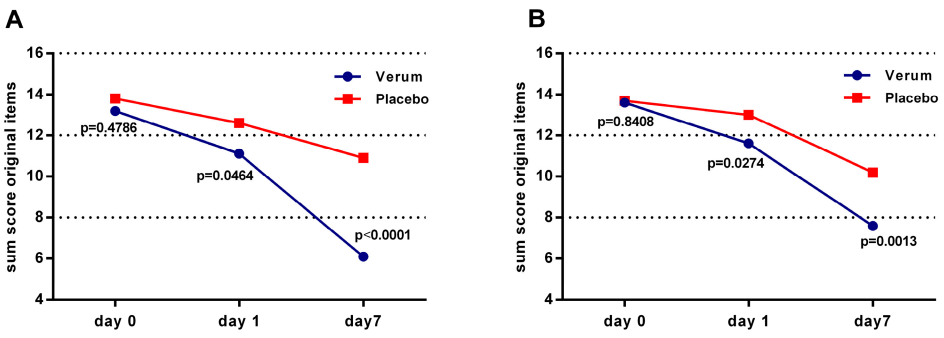

Supplementary Figure 3. Comparison of mean sum-scores of clinical symptoms based on the originally documented symptoms (original items) from Day 0 to Day 7 in the (A) microPP and (B) ITT population.
